# Effects of Ataxia-Telangiectasia Mutated Variants on Radionecrosis and Local Control After Stereotactic Radiation Surgery for Non-Small Cell Lung Cancer Brain Metastases

**DOI:** 10.1016/j.adro.2023.101320

**Published:** 2023-07-22

**Authors:** Warren Floyd, David Carpenter, Eugene Vaios, Rachel Shenker, Peter Hendrickson, Justus D. Adamson, William M. Giles, Chunhao Wang, Karen Allen, Trey Mullikin, Scott R. Floyd, John P. Kirkpatrick, Michelle Green, Zachary J. Reitman

**Affiliations:** a Department of Radiation Oncology; b Department of Pathology, Duke University Medical Center, Durham, North Carolina

## Abstract

**Purpose:**

Genetic variants affecting the radiation response protein ataxia-telangiectasia mutated (ATM) have been associated with increased adverse effects of radiation but also with improved local control after conventional radiation therapy. However, it is unknown whether ATM variants affect rates of radionecrosis (RN) and local intracranial progression (LIP) after stereotactic radiosurgery (SRS) for brain metastases.

**Methods and Materials:**

Patients undergoing an initial course of SRS for non-small cell lung cancer (NSCLC) brain metastases at a single institution were retrospectively identified. Kaplan-Meier estimates were calculated and Cox proportional hazards testing was performed based on ATM variant status.

**Results:**

A total of 541 patients completed SRS for brain metastasis secondary to NSCLC, of whom 260 completed molecular profiling. Variants of ATM were identified in 36 cases (13.8%). Among patients who completed molecular profiling, RN incidence was 4.9% (95% CI, 1.6%-8.2%) at 6 months and 9.9% (95% CI, 4.8%-15.0%) at 12 months. Incidence of RN was not significantly increased among patients with ATM variants, with an RN incidence of 5.3% (95% CI, 0.0%-15.3%) at both 6 and 12 months (*P* = .46). For all patients who completed genomic profiling, LIP was 5.4% (95% CI, 2.4%-8.4%) at 6 months and 9.8% (5.5%-14.1%) at 12 months. A significant improvement in LIP was not detected among patients with ATM variants, with an LIP incidence of 3.1% (0.0%-9.1%) at both 6 and 12 months (*P* = .26). Although differences according to ATM variant type (pathologic variant or variant of unknown significance) did not reach significance, no patients with ATM pathologic variants experienced LIP.

**Conclusions:**

We did not detect significant associations between ATM variant status and RN or LIP after SRS for NSCLC brain metastases. The current data set allows estimation of patient cohort sizes needed to power future investigations to identify genetic variants that associate with significant differences in outcomes after SRS.

## Introduction

Ataxia-telangectasia (A-T) is a rare autosomal recessive disorder characterized by cerebellar degeneration, gonadal and thymic dystrophy, predisposition to cancer, and increased radiation sensitivity.^1^ Study of this disease led to the discovery of the ataxia-telangectasia mutated (ATM) protein, which is altered by a biallelic loss-of-function mutation in patients with A-T. The ATM protein functions as a master regulator of DNA double-strand-break repair and associated stress responses. Mechanistically, ATM is recruited to double-stranded DNA breaks by the MRN (MRE11, RAD50, and NBS1) complex, where it is converted to its active form and initiates signaling cascades regulating DNA damage repair, cell cycle arrest, and autophagy.^2^^,^^3^

The importance of ATM in the maintenance of genomic fidelity and the integrity of the DNA damage repair process suggested that this protein might serve as a tumor suppressor. Subsequent sequencing efforts confirmed this, showing that the ATM gene is mutated in approximately 7% of human tumors assayed.^4^^,^^5^ Interestingly, the radiosensitivity demonstrated in patients with A-T who have characteristic germline ATM loss-of-function mutations was also seen in tumors harboring ATM mutations.^6^ In fact, the loss of ATM function was shown to be associated with improved local control after ionizing radiation therapy in multiple cancer types, including glioma and breast and prostate cancer.^7^, ^8^, ^9^ Additionally, some studies have shown that ATM modulates inflammatory and innate immune processes, raising the possibility that somatic ATM mutations could alter normal-tissue toxicity after radiation therapy.^10^^,^^11^

Alterations in ATM occur frequently in non-small cell lung cancer (NSCLC) and have been shown to be associated with improved local control in this cancer type.^9^ Non-small cell lung cancer is the most common primary histology observed in brain metastases, for which stereotactic radiation therapy (SRS) represents a standard of care for a majority of patients.^12^ As has been observed in primary NSCLC, ATM mutations are common among NSCLC brain metastases.^13^ Despite the relatively high frequency of ATM-mutant NSCLC brain metastases, little is known about the effect of ATM on therapeutic response across ablative radiation therapy doses, particularly in the context of SRS.

Recently, the rapid proliferation of comprehensive genomic profiling (CGP) for human tumors has provided opportunities to inform prognosis and guide therapeutic approaches. In primary NSCLC in particular, key mutations in genes such as *EGFR, BRAF, ROS1, MET, RET, ALK*, and *KRAS* have enabled molecularly targeted therapies.^14^^,^^15^ As the remarkable advances in systemic therapy for patients with NSCLC continue to prolong survival, it will become increasingly important to maximize durable local intracranial control and minimize late radionecrosis (RN) risk for patients with NSCLC brain metastases. For our study, we hypothesized that ATM variants might be associated with increased RN risk as well as increased local control after an initial course of SRS for brain metastases in the setting of NSCLC.

## Methods

### Patients and institutional review board protocols

For this institutional review board–approved retrospective review, we identified all patients with NSCLC who completed an initial SRS course for brain metastases at our institution between January 2015 and December 2020. Patients were cross-referenced with the Institutional Molecular Registry of Tumors, an internally developed data warehouse solution that stores comprehensive genomic testing results.^16^ Cross-referencing of patient outcome and Institutional Molecular Registry of Tumors data was done under Duke University Health System protocol Pro00108074 and was declared exempt from institutional review board review on March 18, 2021. Classification of an ATM variant as a pathogenic variant (PV) or a variant of unknown significance (VUS) was determined per commercially available CGP testing. Exclusion criteria included leptomeningeal carcinomatosis or history of prior SRS. Collected data included demographic variables, clinical characteristics, and treatment parameters.

### Treatments

All patients completed single or multifraction SRS per institutional protocol as previously described,^17^^,^^18^ generally in accordance with Radiation Therapy Oncology Group 90-05 dosing guidelines.^19^ For linear accelerator–based image-guided SRS, patients were simulated in the supine position using a frameless stereotactic mask for immobilization. Gross tumor volume was defined on T1 contrast–enhanced thin-sliced axial magnetic resonance imaging (typically an axial 3-dimensional spoiled gradient with a 1-mm thickness), fused with treatment planning axial computed tomography images (1-mm thickness). Radiosurgery was performed using a Varian TrueBeam STx linear accelerator (Varian, Palo Alto, California) with daily cone beam computed tomography and a 6-degrees-of-freedom motion couch. After SRS completion, patients were followed up with surveillance magnetic resonance of the brain, obtained every 2 to 3 months.

### Statistics

For patient and tumor characteristics, continuous and categorical data were compared using the Wilcoxon rank-sum test and χ^2^ test, respectively. Survival outcomes were measured from the time of SRS completion to the date of death or last follow-up via the Kaplan-Meier method. Cox regression models were used to estimate all hazard ratios for freedom from local intracranial progression (FFLIP; ie, freedom from metastatic progression within “local” target volumes of an initial SRS course) and freedom from RN (FFRN). The LIP and RN events were both determined by multidisciplinary clinical consensus, which incorporated histopathologic diagnosis, response to steroids, and findings from serial magnetic resonance brain scans. Upon review of serial data, both the LIP and the RN events were backdated to the time of initial radiographic concern at 1 or more sites within target volumes of the initial SRS course. In patients undergoing multiple SRS courses, LIP and RN events were restricted to brain metastases treated during the initial SRS course. To account for competing risks, FFRN was censored at the time of initial distant intracranial progression. Where indicated, multiple hypothesis testing was performed via the Benjamini-Hochberg procedure. Analysis was performed using R software, version 4.1.2 (Vienna, Austria).

### Sample size estimates

To estimate the number of patients needed to detect significant differences in the relative hazard (RH) of RN associated with variants in a given gene, a sample-size formula for the proportional-hazards regression model was used.^20^ A given gene of interest has been denoted as gene A. The proportion of patients with gene A variants was set as q_1_. The proportion of patients without gene A variants was set as q_0_, which is equal to 1 – q_1_.

First, the number of events (N) needed to detect a given RH for gene A variant frequency q_1_ was expressed as a function. A 2-tailed α of .05 was set for a threshold probability of rejecting the null hypothesis of no differences in RH between groups (ie, a type I error of concluding that there was a statistically significant difference in RH for RN associated with gene A variants when in reality, the difference came about purely by chance). Z_α_, the standard normal deviate for α associated with α = .05, was 1.96. A β of .2 (power of 80%) was set for the probability of failing to reject the null hypothesis under the alternative hypothesis (ie, the type II error of concluding that there was no significant difference in RH for RN associated with gene A variants when in reality, a difference existed). Z_β_, the standard normal deviate for β associated with β = .2, was 0.8416.

$$A={\left(Z_{\alpha }+Z_{\beta }\right)}^{2}$$

$$B={\left(\log\left[\text{RH}\right]\right)}^{2}\left(1-q_{1}\right)q_{1}$$

$$N=A/B={\left(Z_{\alpha }+Z_{\beta }\right)}^{2}/{\left(\text{log}\left[\text{RH}\right]\right)}^{2}\left(q_{0}\right)q_{1}$$

$$N=7.8489/{\left(\text{log}\left[\text{RH}\right]\right)}^{2}\left(1-q_{1}\right)q_{1}$$

Second, the function was modified to transform the number of events needed (N) to the total number of patients needed (n). This was done by estimating the cumulative incidence for each group, taking into account the censoring rate (CR), median survival time in group 0 (ST_0_), follow-up (FU) rate, and baseline RN event rates for group 0 (BER_0_).^21^ For RN, BER_0_ = 0.0108 events per month, CR = 0.0742 per month, FU = 6.6 months, and ST_0_ = 64.18 months, were estimated based on the current post-SRS patient outcomes data set. For LIP, BER_0_ = 0.0118 events per month, CR = 0.0749 per month, FU = 16.7 months, and ST_0_ = 58.7 months, were estimated based on the current post-SRS patient outcomes data set.

## Results

### Characteristics of patients with NSCLC brain metastasis treated with SRS with or without comprehensive genomic profiling

A total of 541 patients with brain metastases from an NSCLC primary histology completed SRS between 2015 and 2020, of whom 260 completed CGP testing. Comprehensive genomic profiling was performed on cell free-circulating tumor DNA (61%), primary tumor (17%), extracranial metastasis (17%), and brain tumor (18%), including ≥2 sites for 33 patients (13%). The 260 patients with molecular testing were significantly more likely to have a later date for initial brain metastases diagnosis (not tested, 27%; tested, 67% for 2018-2020; *P* < .001) and to be younger at the time of SRS (not tested, 68.0 years; tested, 65.0 years; *P* = .037). The availability of CGP data was not associated with neurosurgical resection before SRS (not tested, 24%; tested, 19%; *P* = .39) (Table 1).

**Table 1:** Demographics and clinical characteristics for all patients at the time of SRS, across comprehensive genomic profiling status and ATM mutational status

Characteristic	Patients*						
	Not tested (n = 281)	Tested (n = 260)	*P* value	ND (n = 224)	VUS (n = 23)	PV (n = 13)	*P* value
Year of SRS completion							
2015-2017	206 (73)	87 (33)	<.001	76 (34)	6 (26)	5 (38)	.69
2018-2020	75 (27)	173 (67)		148 (66)	17 (74)	8 (62)	
Age at time of SRS, median (IQR), y	68 (60-73)	65 (57-73)	.037	64 (57-73)	67 (64-70)	74 (66-80)	.017
Sex							
Male	148 (53)	126 (48)	.33	110 (49)	11 (48)	5 (38)	.76
Female	133 (47)	134 (52)		114 (51)	12 (52)	8 (62)	
Race							
African American	60 (21)	59 (23)	.36	50 (22)	7 (30)	2 (15)	-
White	208 (74)	182 (70)		156 (70)	15 (65)	11 (85)	
Other or unknown	13 (5)	19 (7)		18 (8)	1 (4)	0 (0)	
Tobacco history at time of SRS							
Never	28 (10)	58 (22)	<.001	55 (25)	1 (4)	2 (15)	.07
Yes	252 (90)	202 (78)		169 (75)	22 (96)	11 (85)	
KPS at time of SRS							
100-90	128 (46)	121 (47)	.32	107 (48)	9 (39)	5 (38)	-
80-70	120 (43)	111 (43)		93 (42)	11 (48)	7 (54)	
≤60	27 (10)	17 (7)		13 (6)	3 (13)	1 (8)	
Unknown	6 (2)	11 (4)		11 (5)	0 (0)	0 (0)	
NSCLC histology							
Adenocarcinoma	205 (73)	218 (84)	.002	186 (83)	19 (83)	13 (100)	.27
Not adenocarcinoma	76 (27)	42 (16)		38 (17)	4 (17)	0 (0)	
Brain metastases at time of initial SRS, No.							
1	145 (52)	112 (43)	.06	94 (42)	12 (52)	6 (46)	-
2-5	109 (39)	108 (42)		94 (42)	10 (42)	4 (31)	
6-10	19 (7)	33 (13)		30 (13)	0 (0)	3 (23)	
>10	8 (3)	7 (3)		6 (3)	1 (4)	0 (0)	
Planned target volume of all brain metastases, median (IQR), cm^3^	4.46 (1.38-17.2)	4.74 (1.10-23.3)	.139	4.42 (1.10-22.0)	22.8 (4.77-31.0)	2.43 (0.80-6.67)	.223

*Abbreviations:* IQR = interquartile range; KPS = Karnofsky performance status; ND = not detected; NSCLC = non-small cell lung carcinoma; PV = pathogenic variant; SRS = stereotactic radiosurgery; VUS = variant of unknown significance.

⁎ Data are presented as the number (percentage) of patients unless otherwise indicated.

Additionally, available molecular testing was associated with less frequent tobacco use (any pre-SRS tobacco use: not tested, 90%; tested, 78%; *P* < .001) and adenocarcinoma histology (not tested, 73%; tested, 84%; *P* = .002) (Table 1). Total planned target volume did not differ significantly across ATM status. (Table 1).

### Frequency of ATM alterations among patients with NSCLC brain metastasis who received SRS

Among patients completing molecular testing (n = 260), a total of 36 patients (13.8%) were found to have ATM variants, of which 13 were PV. Patients with ATM variants were significantly older at time of brain metastasis diagnosis (not detected: median, 63.8 years; PV: 73.5 years; *P* = .017), and there was a trend toward an increased rate of smoking (not detected, 75%; PV, 85%; *P* = .07) (Table 1). Among all samples assayed via molecular testing (n = 260), ATM variants were detected in 15 of 159 circulating tumor cell DNA samples (9%), versus in 21 of 101 solid tumor biopsies (21%) (*P* = .010).

There were no significant differences in receipt of cytotoxic chemotherapy, immunotherapy, or targeted therapy, either before or after SRS, between patients with and without ATM variants (Table 2). Additionally, there were no significant differences between groups with respect to rates of *EGFR, ALK, ROS*, or *ERBB2* (Her2) alterations and PDL1 expression levels (Table 3).

**Table 2:** Treatment characteristics for all patients across comprehensive genomic profiling status and ATM variant status

Characteristic	Patients*						
	Not tested (n = 281)	Tested (n = 260)	*P* value	ND (n = 224)	VUS (n = 23)	PV (n = 13)	*P* value
Time from brain Met Dx to any brain Met therapy, mean (IQR), d	14.0 (7.5-21.0)	14.5 (9.0-20.8)	.46	15.0 (9.0-21.0)	15.0 (12.0-23.5)	10.0 (5.0-14.0)	.26
Local intracranial therapy within 2 mo of brain Met Dx							
Resection	68 (24)	49 (19)	.39	34 (15)	13 (57)	2 (15)	-
WBRT	14 (5)	15 (6)		15 (7)	0 (0)	0 (0)	
SRS-FSRT	257 (91)	239 (92)		205 (92)	23 (100)	11 (85)	
None	19 (7)	13 (5)		11 (5)	0 (0)	2 (15)	
Any salvage intracranial therapy							
Yes	99 (35)	129 (50)	.008	107 (48)	14 (61)	8 (62)	.61
No	182 (65)	131 (50)		117 (52)	9 (39)	5 (38)	
Any pre-SRS chemotherapy							
Yes	104 (37)	79 (30)	.10	68 (30)	8 (35)	3 (23)	.76
No	177 (63)	181 (70)		156 (70)	15 (65)	10 (77)	
Any post-SRS chemotherapy							
Yes	90 (32)	108 (42)	.022	95 (42)	8 (35)	5 (38)	.75
No	191 (68)	152 (58)		129 (58)	15 (65)	8 (62)	
Any pre-SRS immunotherapy							
Yes	29 (10)	39 (15)	.10	33 (15)	4 (17)	2 (15)	.94
No	252 (90)	221 (85)		191 (85)	19 (83)	11 (85)	
Any post-SRS immunotherapy							
Yes	90 (32)	124 (48)	<.001	110 (49)	10 (43)	4 (31)	.4
No	191 (68)	136 (52)		114 (51)	13 (57)	9 (69)	
Any pre-SRS targeted therapy							
Yes	10 (4)	20 (8)	.035	16 (7)	3 (13)	1 (8)	.6
No	271 (96)	240 (92)		208 (93)	20 (87)	12 (92)	
Any post-SRS targeted therapy							
Yes	33 (12)	62 (24)	<.001	57 (25)	4 (17)	1 (8)	.26
No	248 (88)	198 (76)		167 (75)	19 (83)	12 (92)	

*Abbreviations:* ATM = ataxia-telangiectasia mutated; Dx = diagnosis; FSRT = fractionated stereotactic radiation therapy; IQR = interquartile range; KPS = Karnofsky performance status; Met = metastases; ND = not detected; PV = pathogenic variant; NSCLC = non-small cell lung carcinoma; SRS = stereotactic radiosurgery; VUS = variant of unknown significance; WBRT = whole brain radiation therapy.

⁎ Data are presented as the number (percentage) of patients unless otherwise indicated.

**Table 3:** Molecular characteristics for patients across ATM variant status

Characteristic	Patients						
	Not tested (n = 281)	Tested (n = 260)	*P* value	ND (n = 224)	VUS (n = 23)	PV (n = 13)	*P* value
PDL-1 status, %							
>49	43 (15)	52 (20)	-	46 (21)	4 (17)	2 (15)	-
1-49	33 (12)	53 (20)		50 (22)	2 (9)	1 (8)	
0	46 (16)	69 (27)		56 (25)	9 (39)	4 (31)	
Unknown or not performed	159 (57)	86 (33)		72 (32)	8 (35)	6 (46)	
*EGFR* mutation or overexpression							
Yes	-	60 (23)	-	53 (24)	4 (17)	3 (23)	.79
No	-	200 (77)		171 (76)	19 (83)	10 (77)	
*ALK* mutation or overexpression							
Yes	-	11 (4)	-	9 (4)	1 (4)	1 (8)	.81
No	-	249 (96)		215 (96)	22 (96)	12 (92)	
*ROS1* mutation or overexpression							
Yes	-	4 (2)	-	4 (2)	0 (0)	0 (0)	.72
No	-	256 (98)		220 (98)	23 (100)	13 (100)	
*HER2* mutation or overexpression							
Yes	-	9 (3)	-	7 (3)	1 (4)	1 (8)	.66
No	-	251 (97)		217 (97)	22 (96)	12 (92)	

*Abbreviations:* ATM = ataxia-telangiectasia mutated; ND = not detected; PV = pathogenic variant; VUS = variant of unknown significance.

### Effects of ATM status on LIP after SRS

**Figure 1:**
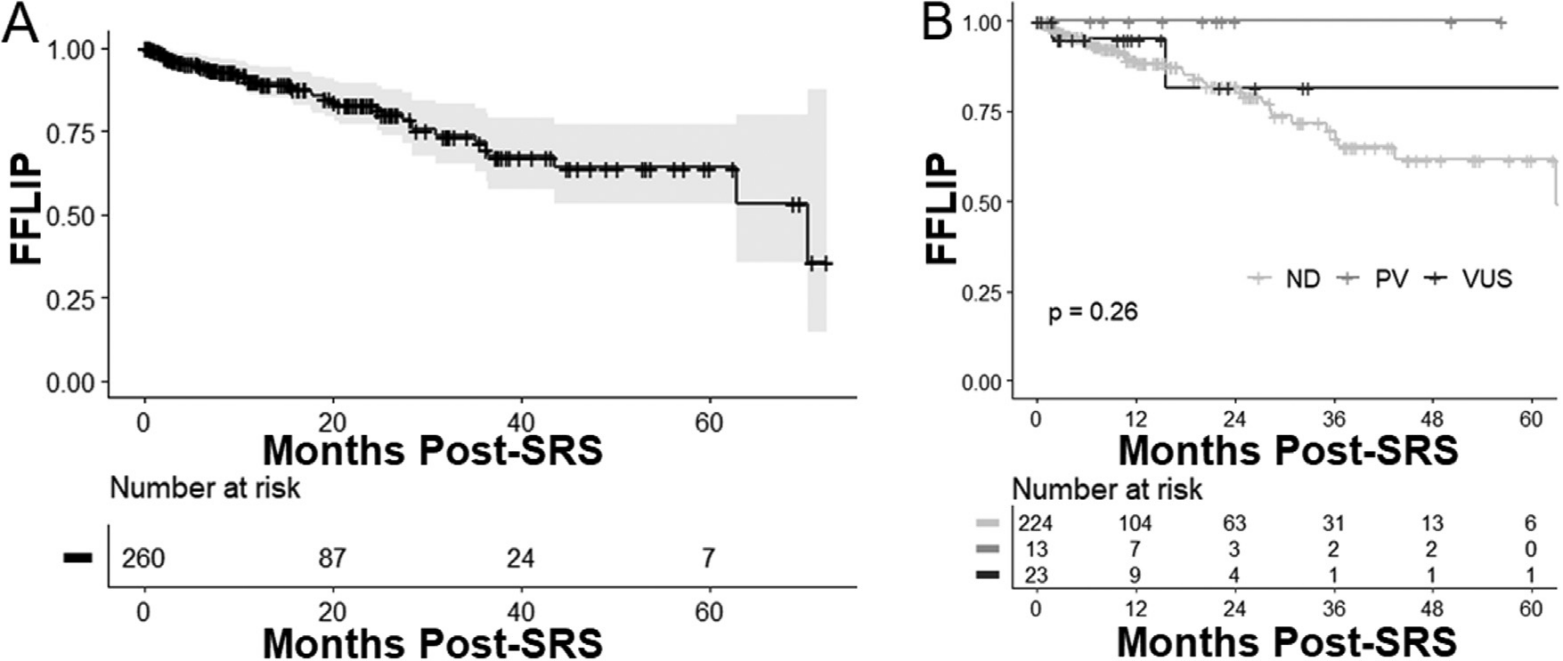
Freedom from local intracranial progression (FFLIP) (A) for all patients with comprehensive genomic profiling and (B) by ATM variant status. The gray shaded area in part A is the 95% CI. *Abbreviations:* ATM = ataxia-telangiectasia mutated; ND = ATM variant not detected; PV = pathogenic ATM variant; SRS = stereotactic radiosurgery; VUS = ATM variant of unknown significance.

Median follow-up from time of SRS completion for patients with and without CPG was 11.3 months (interquartile range [IQR], 6.6-25.2 months) and 7.8 months (IQR, 3.3-22.7 months), respectively. For all patients with CPG, LIP incidence was 5.4% (95% CI, 2.4%-8.4%) at 6 months and 9.8% (5.5%-14.1%) at 12 months (Fig. 1A). For patients with ATM variants of any type (n = 36), LIP incidence was 3.1% (0.0%-9.1%) at both 6 and 12 months, with 1 total LIP event at 2.0 months after SRS. No local intracranial recurrences were observed among patients with pathogenic variants of ATM. However, even when stratified according to variant type (PV vs VUS), ATM variant status was not associated with a significant difference in LIP (*P* = .26) (Fig. 1B). Despite the lack of overall significance, there were no local intracranial recurrences observed among patients with pathogenic variants of ATM.

### Effects of ATM status on RN after SRS

**Figure 2:**
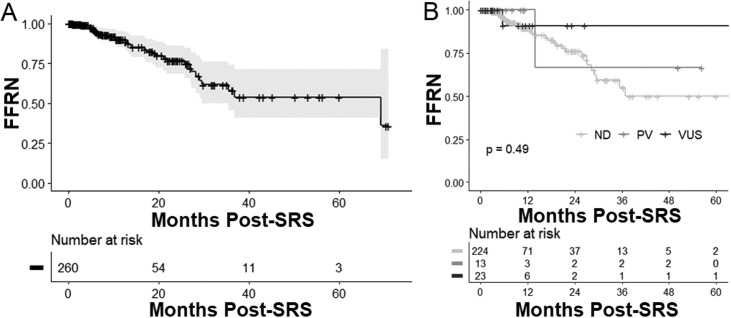
Freedom from radionecrosis (FFRN) (A) for all patients with comprehensive genomic profiling and (B) by ATM variant status. The gray shaded area in part A is the 95% CI. *Abbreviations:* ATM = ataxia-telangiectasia mutated; ND = ATM variant not detected; PV = pathogenic ATM variant; SRS = stereotactic radiosurgery; VUS = ATM variant of unknown significance.

Among patients with CPG, median follow-up was 11.5 months (IQR, 5.0-25.9 months) for those without a detectable ATM variant, 15.3 months (IQR, 6.6-22.4 months) for those with an ATM PV, and 10.7 months (IQR, 3.7-8.9 months) for those with an ATM VUS. For all patients with CPG, RN incidence was 4.9% (95% CI, 1.6%-8.2%) at 6 months and 9.9% (4.8%-15.0%) at 12 months (Fig. 2A). For patients with ATM variants (n = 36), RN incidence was 5.3% (0.0%-15.3%) at both 6 and 12 months, with 2 total RN events, at 5.7 months and 13.9 months after SRS, respectively. Even when stratified according to variant type (pathologic variant vs variant of unknown significance), ATM variant status was not associated with a significant difference in RN (Fig. 2B).

### Estimation of cohort sizes needed to detect genetic variants that significantly affect RN or LIP rates after SRS

To contextualize the statistical power of these findings, we also tested whether any of the 10 most frequent mutations in our cohort resulted in a significant change in LIP or RN. The mutations included *TP53, KRAS, EGFR, CDKN2A, STK11, NF1, ARID1A, ATM, KEAP1*, and *MET*. After correction for multiple hypothesis testing (ie, Benjamini-Hochberg Q values), no variants were found to be associated with a significant difference in either LIP or RN (Table E1).

**Figure 3:**
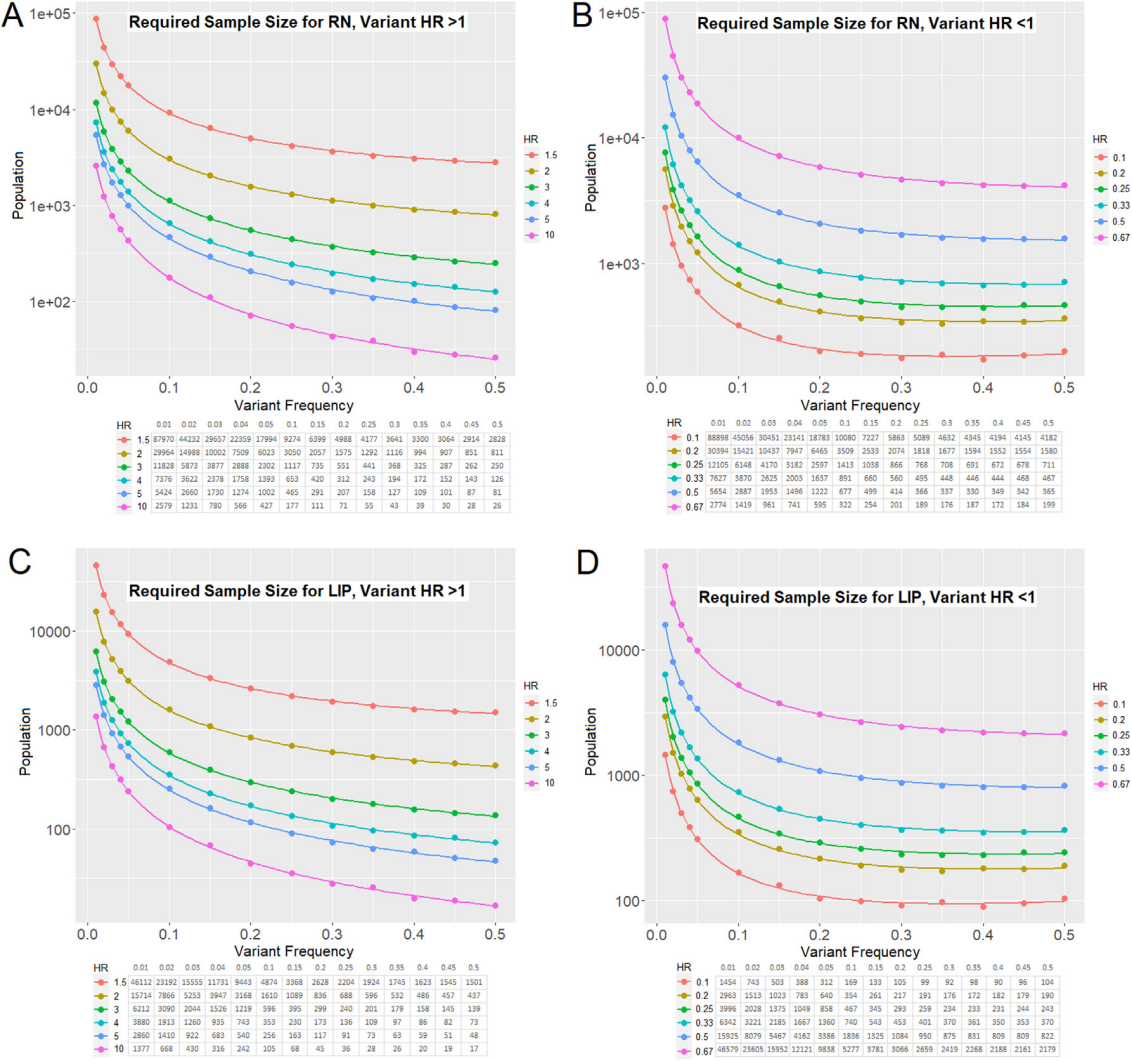
Event rates among patients completing comprehensive genomic profiling were used to estimate the number of patients needed to adequately power analyses of (A, B) RN and (C, D) LIP across a range of hazard ratios and genetic variant frequencies. A and C estimate numbers of patients needed to detect increases in RN and LIP, respectively, whereas B and D estimate numbers of patients needed to detect decreases in these outcomes. *Abbreviations:* LIP = local intracranial progression; RN = radionecrosis.

Future analyses using larger cohorts of patients with available CGP information will be needed to detect significant effects of genetic variants on RN and LIP risk. To guide these analyses, we used the event rates from our current data set to carry out sample-size estimates for such studies (described in the Materials and Methods section). Using the event rates for RN in our study cohort, we plotted the number of patients needed to appropriately power future studies to detect a significantly increased risk of RN associated with a variant of interest (Fig. 3A). We also estimated the number of patients needed to detect a decreased risk of RN (Fig. 3B). Similar approaches were used to estimate the number of patients needed to detect a significantly increased (Fig. 3C) or decreased (Fig. 3D) risk of LIP. Thus, using the observed 14% ATM variant frequency (PV and/or VUS) and hazard ratio of 0.5, we estimate that approximately 1400 patients with NSCLC who have brain metastases would be required to detect a statistically significant difference in LIP after SRS. The same analysis, if limited to a comparison between the presence versus absence of pathogenic ATM variants (5% variant frequency), would require approximately 3400 patients with NSCLC. Given the present cohort size of 260 patients with CPG, we determined that a more than 10-fold reduction in the hazard ratio would be required to detect a significant association between pathogenic ATM variants and local control.

## Discussion

In this study, we report ATM variant incidence in an NSCLC population undergoing SRS for treatment of brain metastases. The ATM variants detected on clinical molecular profiling tests did not predict increased risk of RN. No recurrence was seen among patients with pathologic variants, although this difference was not statistically significant. We also assessed local control, but with the limited number of ATM variants observed in this study, it is difficult to interpret the clinical significance of the numerically improved local control seen in patients with pathogenic ATM variants.

Non-small cell lung cancer remains the leading cause of cancer-related disease, and 25% to 50% of these patients develop brain metastases during the course of their disease.^22^ Identification of genomic biomarkers that inform response to radiation therapy and SRS is of significant clinical interest. Interestingly, somatic ATM mutations are present in approximately 7% of patients with NSCLC.^4^^,^^5^ The ATM protein is a key determinant of tumor radiation response for multiple types of cancer, plays a central role in mediating inflammatory signaling, and is essential in regulating DNA damage response and radioresistance.^10^^,^^11^^,^^13^ Recently, studies demonstrated that ATM functional status alters treatment response. Preclinical data and retrospective analyses, respectively, suggest that ATM deletions radiosensitize primary gliomas to radiation therapy and that ATM variants may predict local intracranial control after conventional radiation therapy.^7^^,^^23^

Despite promising data highlighting the potential for ATM variants to radiosensitize tumors and alter immune response, data on NSCLC radiosensitivity are limited, and results are conflicting. One analysis reported significantly worse disease-free survival among patients with NSCLC with ATM single-nucleotide polymorphisms who were receiving radiation therapy.^24^ In contrast, a pan-cancer study examining the effect of ATM variants on radiation therapy response observed a trend toward improved progression-free survival among patients with lung cancer who had loss-of-function ATM mutations (hazard ratio 0.49, [95% CI, 0.23-1.04]).^13^

To our knowledge, the current study is the first to evaluate the effect of ATM variants on treatment response or toxicity of NSCLC brain metastases receiving SRS. In this study, we questioned whether ATM variants confer an increased rate of RN in patients with NSCLC brain metastasis. Our findings showed no evidence of increased risk of RN for patients with NSCLC brain metastasis with ATM variants versus for patients without detectable ATM variants. Although more data are needed, our first-in-class report suggests that at this time, there is no contraindication for SRS in patients with NSCLC brain metastasis with an ATM variant seen on a clinical genomic profiling test. As molecular profiling enters the standard of care for patients with NSCLC, accurate interpretation and utilization of molecular data to guide clinical decision-making is essential.

We also studied the effect of ATM variants on LIP and were unable to detect a significant difference in recurrence rates between patients bearing tumors with or without detectable ATM variants. Despite the lack of a significant difference in LIP incidence across ATM genotypes in our study, it is notable that no local recurrences were recorded among patients with pathogenic ATM variants. However, it is possible that the failure to detect a significant difference across ATM genotypes for this study can be explained by an insufficient sample size. Although we initially estimated that our cohort size was powered to detect a 4-fold difference in event rates, prior analyses suggest that differences in local control across patients with versus without ATM variants, albeit for conventionally fractionated radiation therapy, are likely closer to a 2-fold (ie, 0.5) hazard ratio.^13^ In addition to contextualizing our findings, these insights inform estimates of appropriately powered cohort sizes for future studies examining the effect of specific mutations on NSCLC brain metastasis radiosensitivity.

This study leveraged clinical molecular profiling tests that are ordered for NSCLC with increasing frequency to guide targeted therapy approaches. The nature of these tests imposes several limitations on the current study. First, clinical molecular profiling tests usually cannot distinguish somatic ATM variants from germline variants. Both types of ATM alterations could potentially influence tumor control, whereas germline variants found in normal tissues are thought to play a more important role in the pathogenesis of RN compared with tumor cell ATM variants. Second, we cannot rule out that ATM variants found in circulating tumor cell DNA profiles could be related to clonal hematopoiesis rather than mutations in the tumor of interest.^25^ However, the majority of ATM variants (59%) were detected via solid tumor biopsies. Contribution from hematopoiesis-related ATM variants likely was not the main determinant of our findings. Our study was not sufficiently powered to determine whether a variant allele fraction or tumor biopsy site (brain metastasis vs primary tumor vs extracranial metastasis) influences the effects of ATM variants on post-SRS outcomes. Correlations between biopsy tissue type (ie, liquid biopsy versus solid tissue) and rates of LIP and RN were not examined. Moreover, tumor heterogeneity presumably depends on timing of tissue sampling with respect to systemic and local therapies, which was not examined. Although the total planned target volume and receipt of systemic therapies before or after SRS did not significantly differ across ATM status, the limited sample size precluded multivariable analysis of ATM status along with known risk factors for LIP and RN, such as intracranial burden, SRS dosimetry parameters such as the volume of brain receiving more than 12 Gy (V12 Gy), and systemic therapy receipt. Moreover, owing to the small sample size, the rates of local progression, radionecrosis, and corresponding power calculations do not account for pre-SRS receipt of whole brain radiation therapy (WBRT) and/or neurosurgical resection nor for corresponding intracranial disease burden at the time of initial diagnosis of brain metastases as opposed to the time of SRS completion. Acknowledging these limitations, this study provides early insights into the possible clinical implications of ATM variants for patients receiving SRS.

## Conclusion

This study is of particular relevance given the recent rapid growth of comprehensive genomic profiling for metastatic NSCLC and the improving overall survival among patients with NSCLC who have brain metastases. Although we failed to observe the large hazard ratios needed to detect significant correlation of ATM variant status with LIP or RN rates, no LIP events were observed among patients with pathogenic ATM variants. Future prospective studies are warranted to validate these findings. In addition, these results provide insight into the frequency and effect size of ATM and other genes of interest, which will be foundational for future clinical trial design.

## Disclosures

Eugene Vaios reported receiving research grants from the National Institutes of Health (NIH) and consulting fees from Glasshouse Health, United Kingdom, and having leadership roles in the American Society for Radiation Oncology Finance/Audit Committee. Scott R. Floyd reported receiving research grants from and/or having contracts with the NIH and the American Cancer Society and having a US patent (9,610,332). John P. Kirkpatrick reported having research grants and/or contracts with SBIR Project Funding, Varian Medical Systems, and BioMimetix JV LLC; receiving consulting fees and speaking honoraria from Monteris Medical; and being the owner and president of Clearsight RT LLC. Michelle Green reported receiving a research grant from Bayer Medical Education and receiving royalties from SciMed Solutions. Zachary J. Reitman reported receiving research grants from and/or having contracts with St. Baldrick's Foundation, the Pediatric Brain Tumor Foundation, the Defeat DIPG Foundation, the SoSo Strong Foundation, the ChadTough Foundation, and the National Cancer Institute; receiving royalties from Genetron Health; receiving payment or honoraria for presentations from Oakstone Publishing and Eisai Pharmaceuticals; and having patents (10711310, 11306364, and 8691960) for brain tumor diagnostic technology and biocatalyst technology, managed by the Duke Office of Licensing and Ventures. All other authors declare no conflicts of interest. None of the organizations or institutions had any role in the collection, analysis, or interpretation of the data, the writing of the manuscript, or the decision to submit it for publication.

## Appendix Supplementary materials

ATM Paper Supplemental Table 1 Image, application 1

## Footnotes

Supplementary material associated with this article can be found in the online version at doi:10.1016/j.adro.2023.101320.
