# Supplementary material for: Effects of Ataxia-Telangiectasia Mutated Variants on Radionecrosis and Local Control After Stereotactic Radiation Surgery for Non-Small Cell Lung Cancer Brain Metastases
Source: Adv Radiat Oncol. 2023 Jul 22;9(1):101320. doi: 10.1016/j.adro.2023.101320 (PMC10801648; doi:10.1016/j.adro.2023.101320)
Supplement: ATM Paper Supplemental Table 1 [file mmc1.docx]

**Supplemental Table 1:** For all patients, the top ten most implicated genetic variants across comprehensive genomic profiling

| **Gene** | **Number of patients** | **Pathogenic Variants** | **Variants of Unknown Significance** | **Local intracranial recurrence-free survival, Q value** | **RN-free survival, p value** |
| --- | --- | --- | --- | --- | --- |
| **TP53** | 149 (57%) | 144 (55%) | 5 (2%) | 0.27 | 0.15 |
| **EGFR** | 66 (25%) | 52 (20%) | 14 (5%) | 0.82 | 0.16 |
| **KRAS** | 61 (23%) | 59 (23%) | 2 (1%) | 0.89 | 0.45 |
| **CDKN2A** | 46 (18%) | 40 (15%) | 6 (2%) | 0.44 | 0.49 |
| **STK11** | 41 (16%) | 32 (12%) | 9 (3%) | 0.3 | 0.57 |
| **NF1** | 38 (15%) | 20 (8%) | 18 (7%) | 0.43 | 0.59 |
| **ARID1A** | 37 (14%) | 12 (5%) | 25 (10%) | 0.63 | 0.69 |
| **ATM** | 36 (14%) | 13 (5%) | 23 (9%) | 0.52 | 0.78 |
| **KEAP1** | 36 (14%) | 15 (6%) | 21 (8%) | 0.53 | 0.84 |
| **MET** | 32 (12%) | 17 (7%) | 15 (6%) | 0.08 | 0.99 |
